# Parvovirus B19 and mumps virus antibodies are major constituents of the intrathecal immune response in European patients with MS and increase the diagnostic sensitivity and discriminatory power of the MRZ reaction

**DOI:** 10.1007/s00415-021-10471-3

**Published:** 2021-03-26

**Authors:** S. Jarius, D. Wilken, J. Haas, K. Ruprecht, L. Komorowski, B. Wildemann

**Affiliations:** 1 Molecular Neuroimmunology Group, Department of Neurology, University of Heidelberg, Heidelberg, Germany; 2 Euroimmun AG, Luebeck, Germany; 3 Department of Neurology, Charité – Universitätsmedizin Berlin, Corporate Member of Freie Universität Berlin, Berlin Institute of Health, Humboldt-Universität zu Berlin, Berlin, Germany

**Keywords:** Multiple sclerosis, MRZ reaction, Polyspecific intrathecal humoral immune response, Parvovirus B19, Mumps virus, Measles virus, Rubella virus, Varicella zoster virus, Herpes simplex virus, Epstein–Barr virus, Cytomegalovirus, Antibody index

## Abstract

**Background:**

A positive MRZ reaction, as defined by intrathecal IgG production against at least two of its constituents, measles virus (M), rubella virus (R) and varicella zoster virus (Z), is detectable in ~ 63% of patients with multiple sclerosis (MS) and is currently considered the laboratory marker with the highest specificity and positive likelihood ratio for MS. However, M, R and Z are only the most well-established constituents of a broader intrathecal humoral immune response in MS.

**Objective:**

To identify additional anti-microbial antibodies inclusion of which in the classical MRZ panel may result in increased sensitivity without compromising the marker’s high specificity for MS.

**Methods:**

We determined the antibody indices (AIs) for 11 viral and bacterial agents (M, R, Z, herpes simplex virus, Epstein–Barr virus, mumps virus, cytomegalovirus, parvovirus B19, *Bordetella pertussis*, *Corynebacterium diphtheriae,* and *Clostridium tetani*) in paired cerebrospinal fluid and serum samples from patients with MS and disease controls.

**Results:**

A positive ‘classical’ MRZ reaction was found in 17/26 (65.4%) MS patients. The five most frequently positive AIs among patients with MS were M (76.9%), Z (61.5%), R (57.7%), parvovirus B19 (42.3%), and mumps (28%). Addition of parvovirus B19 and mumps virus to the MRZ panel resulted in an increase in sensitivity in the MS group from 65.4% to 73.1%, with 22% of the initially MRZ-negative patients exhibiting a de novo-positive response. The extended MRZ panel (‘MRZ*plus*’) distinguished sharply between MS (≥ 3 AIs in 90% of all positives) and controls (varying diagnoses, from migraine to vasculitis; 0-1 AIs; *p *< 0.000001). The highest median AI in the MS group was found for parvovirus B19 (3.97), followed by measles virus (2.79).

**Conclusion:**

Inclusion of parvovirus B19 and mumps virus in the test panel resulted in an increase in the sensitivity and discriminatory power of MRZ. Our results provide a strong rational for prospective studies investigating the role of extended MRZ panels in the differential diagnosis of MS.

## Introduction

Intrathecal production of antibodies to measles virus (M), rubella virus (R) and varicella zoster virus (VZV, Z), the so-called MRZ reaction (MRZR), as defined by the presence of a positive antibody index (AI) to at least two of its three constituents M, R and Z, is the laboratory marker with the highest specificity and positive likelihood ratio (LR) for MS known so far [8, 26]. However, as a limitation, only around 63% of patients with bona fide MS display a positive MRZR [8], resulting in a low negative LR. By contrast, oligoclonal bands (OCBs) are highly sensitive (> 95%) but show only very limited specificity for MS and, accordingly, have a low positive LR and a high negative LR.

The reason for the relatively low sensitivity of the MRZ reaction for MS is poorly understood. However, several studies have shown that the intrathecal humoral immune response in MS may be broader than only M, R and Z and include (non-specific) antibody synthesis against multiple other viral, bacterial or parasitic agents [2, 3, 21, 23, 28, 30]. It is therefore conceivable that the panel of antibody reactivities currently tested (M, R, and Z) may simply be too narrow. Further support for the notion of a positive polyspecific immune reaction being present also in patients who do not meet the classical criteria for a positive MRZR comes from the finding that many MRZR-negative MS patients (i.e., patients who do not display a bi- or trispecific reaction) show at least a positive response to one of its three constituents, mostly to measles virus (despite the fact that there is no evidence for measles being involved in the pathogenesis of MS) [6].

We were therefore interested in whether the inclusion of further anti-microbial antibody indices in the classical MRZ panel would result in a higher sensitivity of the test for MS without compromising the marker’s high specificity. In the present study, we analysed in parallel the intrathecal IgG response to a broad panel of viral and bacterial antigens, including M, R, Z, parvovirus B19 (B), mumps virus (U), HSV1/2 (H), EBV (E; capsid antigen), cytomegalovirus (CMV, C), *Bordetella pertussis* toxin (P), *Corynebacterium diphtheriae* toxin (D) and *Clostridium tetani* toxin (T), in 52 stored matched cerebrospinal fluid (CSF)/serum samples of patients with MS and disease controls.

## Patients and methods

The MS group (median age 48 years [range 16–69]; male:female ratio 1:3.2) consisted of 26 patients with MS according to current McDonald criteria (8 × relapsing remitting MS [RRMS], 10 × secondary progressive MS [SPMS], and 8 × primary progressive MS [PPMS] at the time of lumbar puncture [LP], not treated with steroids before LP per standard operating procedure), while the control group (median age 46 years [range 20–74]; male:female ratio 1:3.3) comprised 26 patients with CNS disorders other than MS (migraine, tension headache, vestibular migraine, vertigo, disorientation, brain tumour, lymphoma, cerebral vasculitis, lupus erythematosus, transient ischemic attack, brain infarction, brain aneurysm, drug-induced headache; no treatment in 25/26, oral steroids in one). Virus-specific antibody levels in CSF and serum were determined using commercially available enzyme-linked immunosorbent assays (ELISAs) (Euroimmun, Lübeck, Germany) according to the manufacturer’s instructions. Total IgG and total albumin concentrations in CSF and serum were determined nephelometrically (BN ProSpec, Siemens Healthcare/Dade Behring, Germany). The intrathecal synthesis of antibodies was detected by calculation of the corresponding anti-microbial AI: AI = Q_IgG[spec]_/Q_IgG[total]_, if Q_IgG[total]_ < Q_lim_, and AI = Q_IgG[spec]_/Q_lim_, if Q_IgG[total]_ > Q_lim_, with Q_IgG[spec]_ = IgG_spec[CSF]_/IgG_spec[serum]_, and Q_IgG[total]_ = IgG_total[CSF]_/IgG_total[serum]_) [26]. The upper reference range of Q_IgG_, Q_lim_, was calculated according to Reiber’s formula [24]:

$$Q_{{\lim \left( {IgG} \right)}} = 0.93\sqrt {\left( {Q_{AIb} } \right)^{2} + 6 \times 10^{ - 6} } - 1.7 \times 10^{ - 3}$$

AI values > 1.5 were considered to be indicative of intrathecal IgG production against the respective pathogen [26]. All samples were stored at −80 °C until testing. The study was approved by the institutional review board of the University of Heidelberg, and patients gave written informed consent. If no written consent could be obtained retrospectively, samples were tested in strictly anonymized fashion as requested by the institutional review board. All samples were tested as part of a larger project on the differential laboratory diagnosis of MS.

## Results

Of the MS patients, 17/26 (65.4%) displayed a positive MRZ reaction, as defined by elevated AIs for at least two of the three viruses M, R and Z. Of these, 13 patients showed intrathecal synthesis against all 3 viruses (M + R + Z; ‘trispecific reaction’) and 4 had elevated AIs for 2 of the viruses (2 × M + R, 2 × M + Z, 0 × R + Z; ‘bispecific reaction’); another 4 patients with MS displayed a monospecific reaction (3 × M, 0 × R, 1 × Z), and 5 had no detectable intrathecal reaction to M, R or Z (Tables 1, 2).

**Table 1:** Frequency of positive antibody indices (AI) for measles virus (M), rubella virus (R), varicella zoster virus (V), herpes simplex virus (H), Epstein Barr virus (E), mumps virus (U), cytomegalovirus (C), parvovirus B19 (B), *Bordetella pertussis* (P), *Corynebacterium diphtheriae* (D) and *Clostridium tetani* (T) in matched CSF/serum pairs from patients with MS and disease controls

AI	MS	Controls	Controls vs. MS
M	20/26 (76.9%)	0/26 (0%)	*p* < 0.000001
R	15/26 (57.7%)	0/26 (0%)	*p* < 0.000001
Z	16/26 (61.5%)	0/26 (0%)	*p* < 0.000001
B	11/26 (42.3%)	0/26 (0%)	*p* < 0.0003
H	1/22 (4.5%)	3/15 (20%)	n.d
E	3/22 (13.6%)	1/24 (4.2%)	n.d
C	0/22 (0%)	2/15 (13.3%)	n.d
U	7/25 (28%)	1/23 (4.3%)^§^	n.d
P	0/22 (0%)	0/15 (0%)	n.d
D	0/22 (0%)	0/15 (0%)	n.d
T	4/22 (18.2%)	1/15 (6.7%)	n.d

^§^Borderline positive result in a single control patient (1.55; cut-off 1.5)

**Table 2:** MRZ, MRZB and MRZBU reaction in matched CSF/serum samples from patients with MS and disease controls

AI panel	MS	Controls	Controls vs. MS
MRZ	17/26 (65.4%)	0/26 (0%)	*p* < 0.000001
M + R + Z	13/26 (50%)	0/26 (0%)	
M + R	2/26 (7.7%)	0/26 (0%)	
M + Z	2/26 (7.7%)	0/26 (0%)	
R + Z	0/26 (0%)	0/26 (0%)	
MRZB	18/26 (69.2%)	0/26 (0%)	*p* < 0.000001
M + R + Z + B	4/26 (15.4%)	0/26 (0%)	
M + R + Z	9/26 (34.6%)	0/26 (0%)	
M + R + B	2/26 (7.7%)	0/26 (0%)	
M + Z + B	2/26 (7.7%)	0/26 (0%)	
R + Z + B	0/26 (0%)	0/26 (0%)	
M + B	1/26 (3.8%)	0/26 (0%)	
M + R	0/26 (0%)	0/26 (0%)	
M + Z	0/26 (0%)	0/26 (0%)	
R + B	0/26 (0%)	0/26 (0%)	
R + Z	0/26 (0%)	0/26 (0%)	
Z + B	0/26 (0%)	0/26 (0%)	
MRZBU	19/25 (76%)	0/23 (0%)	*p* < 0.000001
M + R + Z + B + U	2/25 (8%)	0/23 (0%)	
M + R + Z + B	2/25 (8%)	0/23 (0%)	
M + R + Z + U	1/25 (4%)	0/23 (0%)	
M + R + B + U	1/25 (4%)	0/23 (0%)	
M + Z + B + U	1/25 (4%)	0/23 (0%)	
R + Z + B + U	0/25 (0%)	0/23 (0%)	
M + R + Z	8/25 (32%)	0/23 (0%)	
M + R + B	1/25 (4%)	0/23 (0%)	
M + R + U	0/25 (0%)	0/23 (0%)	
M + Z + B	1/25 (4%)	0/23 (0%)	
M + Z + U	0/25 (0%)	0/23 (0%)	
M + B + U	1/25 (4%)	0/23 (0%)	
R + Z + B	0/25 (0%)	0/23 (0%)	
R + Z + U	0/25 (0%)	0/23 (0%)	
R + B + U	0/25 (0%)	0/23 (0%)	
Z + B + U	0/25 (0%)	0/23 (0%)	
M + R	0/25 (0%)	0/23 (0%)	
M + Z	0/25 (0%)	0/23 (0%)	
M + B	0/25 (0%)	0/23 (0%)	
M + U	0/25 (0%)	0/23 (0%)	
R + Z	0/25 (0%)	0/23 (0%)	
R + B	0/25 (0%)	0/23 (0%)	
R + U	0/25 (0%)	0/23 (0%)	
Z + B	0/25 (0%)	0/23 (0%)	
Z + U	0/25 (0%)	0/23 (0%)	
B + U	1/25 (4%)	0/23 (0%)	

*AI* antibody index, *M* measles virus, *R* rubella virus, *V* varicella zoster virus, *B* parvovirus B19, *U* mumps virus

Of the 17 MS samples positive for the classical MRZR, 12 (71%) were positive for at least one of the additional AIs tested (8 × B [3 × B, 2 × B + U, 1 × B + E + U, 1 × B + H, 1 × B + U + T], 3 × T [2 × T, 1 × E + T], 1 × U), corroborating the notion that the spectrum of the polyspecific humoral immune response in MS is indeed broader than just M, R and Z (median 1.5 additional AIs, range 0–3 in those positive for the classical MRZR) and suggesting that it may particularly frequently include parvovirus B19. As a limitation, four MRZ-positive MS patients could not be tested for all AIs due to a lack of material, which leaves the possibility that the real prevalence of additional positive AIs might even be higher and the spectrum of possible AI combinations even broader than reported here.

Of those MS patients with a negative classical MRZR, three (33%) were positive for one or more of the additional positive AIs, with B again prevailing (1 × B, 1 × B + U, 1 × B + E + U), resulting in a de novo positive (i.e., bi- or trispecific) reaction in two patients (1 × M + B + E + U, 1 × B + U) and thus in an increase in sensitivity in the MS group from 65.4% (17/26) to 73.1% (19/26). By contrast, none of the other additional AIs tested (D, T, P, H, C, E) resulted in an increase in sensitivity.

If all patients with MS are considered, 15/26 (58%) were positive for at least one (median 2 [range 1–3]) of the ‘additional' AIs tested and 23/26 (89%) for at least one of the 11 AIs tested in total (median 4 [range 1–5]); in 3 patients none of the 11 AIs was positive.

As a drawback, inclusion of some of the additional AIs in the panel resulted in a decline in specificity, with four controls positive for at least one of the additional AIs and two of them, who had been MRZ-negative based on the original panel consisting of M, R and Z, displaying  a bi- or trispecific reaction. However, when restricting the panel to MRZ + B + U, a positive (i.e., at least bispecific) reaction was observed in none of the control patients. None of the controls exhibited an intrathecal response to either M, R or Z, corroborating the high specificity of the original panel for MS. The difference between the MS and the control group regarding the frequency of a positive MRZ, MRZB or MRZBU reaction, as defined by a bi- or trispecific response, was highly significant (*p* < 0.00001, Mann–Whitney *U* test, irrespective of whether the single steroid-treated control patient was included or not).

A single (MRZBU-negative) control patient showed an isolated borderline positive AI for mumps (1.55; cut-off 1.5). We therefore tested whether the use of a more conservative cut-off for AI positivity would result in a decrease in sensitivity. When applying a cut-off of 1.6 or 1.7 for all AIs, the sensitivity of the extended MRZB or MRZBU panel remained unaltered and none of the controls exhibited a positive AI for parvovirus B19 or mumps virus (not shown).

Cross-reactivity between *Herpesviridae* is a potential issue, since it would result in false-positive bi- or multispecific responses. Overall, 3/37 (8.1%) patients tested for Z, H, E and C reacted against at least two of these four herpes viruses (2 × MS; 1 × disease control): one Z-AI-positive MS sample showed a positive H-AI and one a positive E-AI; in addition, a single patient with suspected cerebral vasculitis in the control group showed a positive intrathecal response to H, E and C.

Of note, all 4 MS patients who had displayed a bispecific reaction based on the classical MRZ panel showed a trispecific reaction after inclusion of B and U in the diagnostic panel and 17 of all 19 (90%) MRZ*plus*-positive MS patients had at least a trispecific reaction (10 × 3 AIs, 5 × 4 AIs, 2 × 5 AIs), resulting in a more distinct discrimination between controls (0–1 AI) and MS patients (mostly 3 or more AIs).

No significant correlation between AI values and age at the time of LP was found, neither if all AIs nor if only positive AIs are considered. Moreover, the median age did not differ among MRZ*plus*-positive and MRZ*plus*-negative MS patients (48 years in both subgroups).

**Fig. 1:**
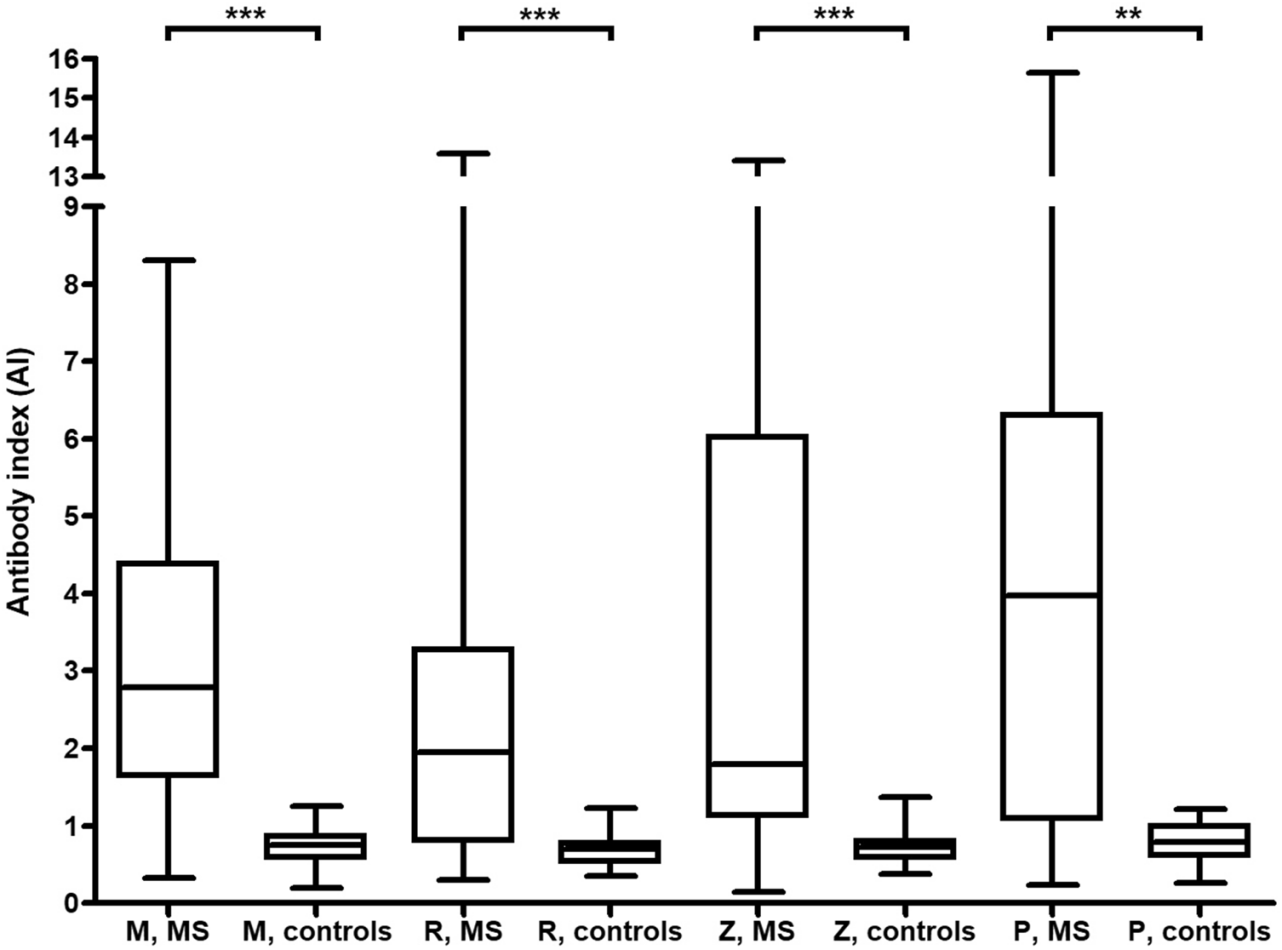
Box plots showing antibody indices for measles virus (M), rubella virus (R), varicella zoster virus (Z) and parvovirus B19 (B) in multiple sclerosis and in disease controls. Groups were compared using the Kruskal–Wallis test. Mumps virus antibodies were not present in a sufficient number of controls to allow meaningful comparisons, and mumps virus AIs are therefore not shown

Median AIs for M, R, Z and B differed significantly between patients with MS and disease controls (*p* < 0.0001; Kruskal–Wallis) (Fig. 1). Of note, the highest median AI in the MS group was found for parvovirus B19 (AI = 3.97), followed by measles virus (AI = 2.79) (Fig. 1).

## Discussion

This study is one of the largest performed so far on the intrathecal antimicrobial immune response in MS. Investigating a panel of eight additional antimicrobial AIs, we identified intrathecally produced antibodies to parvovirus B19 and mumps virus as novel promising markers for MS. Especially, addition of parvovirus B19 to the classical MRZ panel could help to increase the sensitivity of the MRZ reaction without compromising its specificity. Of further note, mumps virus was positive in 7/25 (28%) MS patients but only in a single control patient, who exhibited a borderline result (AI = 1.55; cut-off 1.5) (Table 1), rendering mumps virus another potentially interesting marker. In accordance with the latter finding, Sindic (1998) detected OCBs to M, R, Z and mumps virus in 18/18 patients with MS using an antigen-driven capillary blot technique, 15 of whom (83%) displayed a bi- or trispecific reaction [5, 31]. Applying a slightly more conservative AI cut-off (1.6 or 1.7 instead of 1.5) resulted in 100% specificity of the extended panel (tentatively termed ‘MRZ*plus*') without causing a decline in sensitivity. This should be taken into account in future studies. As the MRZ test is mainly used as a ‘rule-in test’ rather than as a ‘rule-out test’ in MS, high specificity if of utmost importance.

It is of interest that a few of the control patients showed a monospecific intrathecal immune response to herpes viruses: a positive H-AI was noted in two patients with migraine and a positive C-AI in a patient with non-classified “cephalgia”. Although not likely, we cannot formally rule out that headache was related to herpes virus infection in these cases, since no polymerase chain reaction (PCR) was performed and the virus-specific intrathecal IgG fraction, F(s) [7, 21] not determined due to a lack of material. The finding of an intrathecal immune response to several herpes viruses (H, E and C) in a further control patient with suspected cerebral vasculitis corroborates previous concerns about cross-reactivities between *Herpesviridae* (since simultaneous CNS infection with all three viruses is highly unlikely) [4]. Although a response to more than one herpes virus was relatively infrequent in the present study, the risk of cross-reactivity would argue against including more than one herpes virus in the MRZ panel. In any case, in accordance with a previous study [26], inclusion of herpes simplex virus in the panel did not result in an increase in sensitivity in the present study, nor was inclusion of EBV or CMV associated with such an increase. A positive AI for herpes simplex virus was even more common in the control than in the MS group (*N* = 3 vs. *N* = 1), as was a positive AI for CMV, despite the fact that not all controls could be tested for these two AIs due to a lack of material (Table 1).

Our study once more confirms the high specificity of the classical MRZ panel. It also strongly corroborates the notion of the MRZ reaction being just part of a much broader, polyspecific humoral immune response in MS. The latter notion is in accordance with the fact that antibodies to M, R and Z account only for a proportion of intrathecally produced CSF IgG [7] and are not accountable for the majority of CSF oligoclonal IgG bands in MS [31]. However, it should be underlined that there is currently no evidence that any of the viruses that form part of the MRZB or MRZBU reaction is actively involved in the aetiopathogenesis of MS. PCR studies did not demonstrate reactivation of measles virus, rubella virus, zoster virus, parvovirus B19 or mumps virus during acute attacks [6, 19]. This is consistent with the concept that the MRZ reaction represents non-specific (‘nonsense’) B-cell activation. Its exact role in the immunopathophysiology of MS has still to be elucidated.

Of note, MS is strongly associated with EBV, with nearly 100% of patients with MS being seropositive for anti-EBV antibodies. EBV is a B lymphotropic virus, and acute EBV infection is known to lead to a strong polyspecific activation of B cells [33]. It has thus been hypothesized that polyspecific antibody producing B lineage cells may enter the CNS of patients with MS at the time of and triggered by acute EBV infection [20, 29] and that the exact composition of the intrathecally produced repertoire of antimicrobial antibodies in MS may thus mirror the presence or absence of specific B-cell clones at the very time of EBV infection (immunological ‘snapshot’). Given the delay between EBV infection and generation of anti-EBV antibodies, this would mostly include non-EBV-specific B-cell clones, which could explain why a positive EBV-AI is relatively rare in MS—as opposed to a positive AI to M, R, Z and other antigens—despite the fact that virtually all patients with MS are positive for serum antibodies to EBV [29]. The frequency of elevated EBV-AIs in patients with MS observed in the present study (13.6%) is in good accordance with the results of previous studies, which found an elevated EBV AI in in 4.3–15.6%, depending on the EBV antigen used, of adult patients with MS [29]. Of note, contact with M, R, Z, B and U, i.e., the five antigens found to be useful in the present study, usually occurs during early childhood and thus indeed prior to contact with EBV. As expected, all MS patients tested in this study were positive for serum anti-EBV antibodies; by contrast, three disease controls were seronegative.

It should not go unmentioned that other factors than panel composition may influence the frequency of a positive MRZ reaction in a given cohort: (1) It has been shown that the MRZ reaction is virtually absent in important MS mimics such as myelin oligodendrocyte glycoprotein (MOG)-IgG-positive encephalomyelitis (MOG-EM; also termed MOG antibody-related autoimmune disorder, or MOGAD) [13, 15, 16] and aquaporin-4 (AQP4)-IgG-positive neuromyelitis optica spectrum disorder (NMOSD) [10, 14, 17, 32], ADEM[8, 11], paraneoplastic neurological disorders [8, 9] and neuroborreliosis [1, 8]. Accidental inclusion of such patients in studies investigating the MRZR in MS will result in underestimating the marker’s sensitivity. (2) A positive MRZ reaction might be less frequent in children; while this might be due to the difference between prepubertal and postpubertal prevalence rates for rubella virus antibodies [8, 25], it may partly also reflect accidental inclusion of children with MOG-EM—a condition that is common among children with CNS demyelination (and even more common than MS in young children)—in previous pediatric studies. (3) The number of positive AIs and thus the frequency of a bi- or trispecific MRZR increased with disease duration in one study [22]. (4) Differences in MRZR frequency between various MS subtypes may play a role, given that a negative MRZ reaction was found in the few patients with histopathologically defined pattern II or pattern III MS or Baló’s concentric sclerosis analysed so far [12, 18]. These conditions are also much less frequently associated with intrathecal total IgG synthesis (as indicated by negative OCBs and a normal CSF/serum ratio) and thus may represent entities immunopathogenetically different from classical pattern I MS [12, 18].

Parvovirus B19 is not usually considered a ‘neurotropic’ virus. However, it is important to note that the polyspecific intrathecal humoral immune response in MS is not restricted to neurotropic viruses. It has already been shown to include also antibodies to *Chlamydia pneumoniae*, both in adults and children, and to several other microbiological agents that do not typically cause CNS infection [2, 28]. Given that the research on CSF antibodies in MS was originally driven by the interest in a then supposed viral aetiology of MS, the focus on neurotropic viruses in much of the existing literature on the MRZ reaction may have historic reasons. Moreover, the frequency of the classic MRZ reaction has been shown to be linked to the individual vaccination status [27], suggesting that a history of actual CNS infection is not required. The presence of a broader panel of anti-microbial antibody responses that is not restricted to ‘neurotropic’ viruses would be in line with the notion of the MRZ reaction simply reflecting parts of the individual B cell repertoire present at the time of the first EBV infection in patients with MS [20]. 

### Strengths and limitations

The following potential limitations should be mentioned: (1) Some of the ELISAs used for determining AIs (B, D, P and T) in the present study are in-house assays, i.e., they have not yet been officially approved by the marketing authorities for use in CSF analytics. (2) Previous studies on the classical MRZ reaction were mostly conducted using assays manufactured by Dade Behring/Siemens, Germany, while ELISAs manufactured by Euroimmun were employed in the present study. However, regular round-robin tests performed by INSTAND e.V. (www.instand-ev.de) have shown excellent sensitivity and specificity of the assays used here compared with other MRZ assays. (3) Although the control group comprised patients with various inflammatory and non-inflammatory neurological diseases, further studies that include large numbers of patients with relevant differential diagnoses of MS will be necessary to assess the specificity of the extended MRZ panel for MS in a definite way.

## Conclusion

In summary, addition of AIs for parvovirus B19 and mumps to the classical MRZ panel (‘MRZ*plus*’) was associated with an increase in sensitivity for MS without major loss in specificity, while inclusion of AIs to HSV, EBV, CMV, diphtheria, pertussis and tetanus did not result in an increase in sensitivity. Our study provides a rationale for larger, prospective studies on the impact of adding parvovirus B19 and mumps to the MRZ panel. Such studies should include more controls with inflammatory CNS disorders and should ideally be performed prospectively and in a multicentre setting.

## References

[1] Bednarova J, Stourac P, Adam P (2005). Relevance of immunological variables in neuroborreliosis and multiple sclerosis. Acta Neurol Scand.

[2] Derfuss T, Gurkov R, Then Bergh F, Goebels N, Hartmann M, Barz C, Wilske B, Autenrieth I, Wick M, Hohlfeld R, Meinl E (2001). Intrathecal antibody production against Chlamydia pneumoniae in multiple sclerosis is part of a polyspecific immune response. Brain.

[3] Derfuss T, Hohlfeld R, Meinl E (2005). Intrathecal antibody (IgG) production against human herpesvirus type 6 occurs in about 20% of multiple sclerosis patients and might be linked to a polyspecific B-cell response. J Neurol.

[4] Felgenhauer K, Reiber H (1992). The diagnostic significance of antibody specificity indices in multiple sclerosis and herpes virus induced diseases of the nervous system. Clin Investig.

[5] Frederiksen JL, Sindic CJ (1998). Intrathecal synthesis of virus-specific oligoclonal IgG, and of free kappa and free lambda oligoclonal bands in acute monosymptomatic optic neuritis. Comparison with brain MRI. Mult Scler.

[6] Godec MS, Asher DM, Murray RS, Shin ML, Greenham LW, Gibbs CJ, Gajdusek DC (1992). Absence of measles, mumps, and rubella viral genomic sequences from multiple sclerosis brain tissue by polymerase chain reaction. Ann Neurol.

[7] Jacobi C, Lange P, Reiber H (2007). Quantitation of intrathecal antibodies in cerebrospinal fluid of subacute sclerosing panencephalitis, herpes simplex encephalitis and multiple sclerosis: discrimination between microorganism-driven and polyspecific immune response. J Neuroimmunol.

[8] Jarius S, Eichhorn P, Franciotta D, Petereit HF, Akman-Demir G, Wick M, Wildemann B (2017). The MRZ reaction as a highly specific marker of multiple sclerosis: re-evaluation and structured review of the literature. J Neurol.

[9] Jarius S, Eichhorn P, Jacobi C, Wildemann B, Wick M, Voltz R (2009). The intrathecal, polyspecific antiviral immune response: Specific for MS or a general marker of CNS autoimmunity?. J Neurol Sci.

[10] Jarius S, Franciotta D, Bergamaschi R, Rauer S, Wandinger KP, Petereit HF, Maurer M, Tumani H, Vincent A, Eichhorn P, Wildemann B, Wick M, Voltz R (2008). Polyspecific, antiviral immune response distinguishes multiple sclerosis and neuromyelitis optica. J Neurol Neurosurg Psychiatry.

[11] Jarius S, Franciotta D, Marchioni E, Hohlfeld R, Wildemann B, Voltz R (2006). Intrathecal polyspecific immune response against neurotropic viruses discriminates between multiple sclerosis and acute demyelinating encephalomyelitis. J Neurol.

[12] Jarius S, Konig FB, Metz I, Ruprecht K, Paul F, Bruck W, Wildemann B (2017). Pattern II and pattern III MS are entities distinct from pattern I MS: evidence from cerebrospinal fluid analysis. J Neuroinflammation.

[13] Jarius S, Lechner C, Wendel EM, Baumann M, Breu M, Schimmel M, Karenfort M, Marina AD, Merkenschlager A, Thiels C, Blaschek A, Salandin M, Leiz S, Leypoldt F, Pschibul A, Hackenberg A, Hahn A, Syrbe S, Strautmanis J, Hausler M, Krieg P, Eisenkolbl A, Stoffels J, Eckenweiler M, Ayzenberg I, Haas J, Hoftberger R, Kleiter I, Korporal-Kuhnke M, Ringelstein M, Ruprecht K, Siebert N, Schanda K, Aktas O, Paul F, Reindl M, Wildemann B, Rostasy K, in cooperation with the Bsg, the Neuromyelitis optica Study G (2020) Cerebrospinal fluid findings in patients with myelin oligodendrocyte glycoprotein (MOG) antibodies Part 2: Results from 108 lumbar punctures in 80 pediatric patients. J Neuroinflamm 17:262 10.1186/s12974-020-01825-1 PMC7470445 32883358

[14] Jarius S, Paul F, Franciotta D, Ruprecht K, Ringelstein M, Bergamaschi R, Rommer P, Kleiter I, Stich O, Reuss R, Rauer S, Zettl UK, Wandinger KP, Melms A, Aktas O, Kristoferitsch W, Wildemann B (2011). Cerebrospinal fluid findings in aquaporin-4 antibody positive neuromyelitis optica: results from 211 lumbar punctures. J Neurol Sci.

[15] Jarius S, Pellkofer H, Siebert N, Korporal-Kuhnke M, Hummert MW, Ringelstein M, Rommer PS, Ayzenberg I, Ruprecht K, Klotz L, Asgari N, Zrzavy T, Hoftberger R, Tobia R, Buttmann M, Fechner K, Schanda K, Weber M, Asseyer S, Haas J, Lechner C, Kleiter I, Aktas O, Trebst C, Rostasy K, Reindl M, Kumpfel T, Paul F, Wildemann B, in cooperation with the Neuromyelitis Optica Study G (2020). Cerebrospinal fluid findings in patients with myelin oligodendrocyte glycoprotein (MOG) antibodies Part 1: Results from 163 lumbar punctures in 100 adult patients. J Neuroinflammation.

[16] Jarius S, Ruprecht K, Kleiter I, Borisow N, Asgari N, Pitarokoili K, Pache F, Stich O, Beume LA, Hummert MW, Ringelstein M, Trebst C, Winkelmann A, Schwarz A, Buttmann M, Zimmermann H, Kuchling J, Franciotta D, Capobianco M, Siebert E, Lukas C, Korporal-Kuhnke M, Haas J, Fechner K, Brandt AU, Schanda K, Aktas O, Paul F, Reindl M, Wildemann B, in cooperation with the Neuromyelitis Optica Study G (2016) MOG-IgG in NMO and related disorders: a multicenter study of 50 patients. Part 2: Epidemiology, clinical presentation, radiological and laboratory features, treatment responses, and long-term outcome. J Neuroinflammation 13:280 10.1186/s12974-016-0718-0 PMC5086042 27793206

[17] Jarius S, Ruprecht K, Wildemann B, Kuempfel T, Ringelstein M, Geis C, Kleiter I, Kleinschnitz C, Berthele A, Brettschneider J, Hellwig K, Hemmer B, Linker RA, Lauda F, Mayer CA, Tumani H, Melms A, Trebst C, Stangel M, Marziniak M, Hoffmann F, Schippling S, Faiss JH, Neuhaus O, Ettrich B, Zentner C, Guthke K, Hofstadt-van Oy U, Reuss R, Pellkofer H, Ziemann U, Kern P, Wandinger KP, Then Bergh F, Boettcher T, Langel S, Liebetrau M, Rommer PS, Niehaus S, Munch C, Winkelmann A, Zettl UK, Metz I, Veauthier C, Sieb JP, Wilke C, Hartung HP, Aktas O, Paul F (2012). Contrasting disease patterns in seropositive and seronegative neuromyelitis optica: a multicentre study of 175 patients. J Neuroinflammation.

[18] Jarius S, Wurthwein C, Behrens JR, Wanner J, Haas J, Paul F, Wildemann B (2018). Balo's concentric sclerosis is immunologically distinct from multiple sclerosis: results from retrospective analysis of almost 150 lumbar punctures. J Neuroinflammation.

[19] Nakashima I, Fujihara K, Itoyama Y (1999). Human parvovirus B19 infection in multiple sclerosis. Eur Neurol.

[20] Otto C, Hofmann J, Ruprecht K (2016). Antibody producing B lineage cells invade the central nervous system predominantly at the time of and triggered by acute Epstein-Barr virus infection: a hypothesis on the origin of intrathecal immunoglobulin synthesis in multiple sclerosis. Med Hypotheses.

[21] Otto C, Oltmann A, Stein A, Frenzel K, Schroeter J, Habbel P, Gartner B, Hofmann J, Ruprecht K (2011). Intrathecal EBV antibodies are part of the polyspecific immune response in multiple sclerosis. Neurology.

[22] Petereit HF, Reske D (2005). Expansion of antibody reactivity in the cerebrospinal fluid of multiple sclerosis patients - follow-up and clinical implications. Cerebrospinal Fluid Res.

[23] Pohl D, Rostasy K, Jacobi C, Lange P, Nau R, Krone B, Hanefeld F (2010). Intrathecal antibody production against Epstein-Barr and other neurotropic viruses in pediatric and adult onset multiple sclerosis. J Neurol.

[24] Reiber H (1998). Cerebrospinal fluid–physiology, analysis and interpretation of protein patterns for diagnosis of neurological diseases. Mult Scler.

[25] Reiber H, Teut M, Pohl D, Rostasy KM, Hanefeld F (2009). Paediatric and adult multiple sclerosis: age-related differences and time course of the neuroimmunological response in cerebrospinal fluid. Mult Scler.

[26] Reiber H, Ungefehr S, Jacobi C (1998). The intrathecal, polyspecific and oligoclonal immune response in multiple sclerosis. Mult Scler.

[27] Robinson-Agramonte M, Reiber H, Cabrera-Gomez JA, Galvizu R (2007). Intrathecal polyspecific immune response to neurotropic viruses in multiple sclerosis: a comparative report from Cuban patients. Acta Neurol Scand.

[28] Rostasy K, Reiber H, Pohl D, Lange P, Ohlenbusch A, Eiffert H, Maass M, Hanefeld F (2003). Chlamydia pneumoniae in children with MS: frequency and quantity of intrathecal antibodies. Neurology.

[29] Ruprecht K, Wildemann B, Jarius S (2018). Low intrathecal antibody production despite high seroprevalence of Epstein-Barr virus in multiple sclerosis: a review of the literature. J Neurol.

[30] Schubert J, Weissbrich B (2007). Detection of virus-specific intrathecally synthesised immunoglobulin G with a fully automated enzyme immunoassay system. BMC Neurol.

[31] Sindic CJ, Monteyne P, Laterre EC (1994). The intrathecal synthesis of virus-specific oligoclonal IgG in multiple sclerosis. J Neuroimmunol.

[32] Sven Jarius, Klemens Ruprecht, Ingo Kleiter, Nadja Borisow, Nasrin Asgari, Kalliopi Pitarokoili, Florence Pache, Oliver Stich, Lena-Alexandra Beume, Martin W. Hümmert, Corinna Trebst, Marius Ringelstein, Orhan Aktas, Alexander Winkelmann, Mathias Buttmann, Alexander Schwarz, Hanna Zimmermann, Alexander U. Brandt, Diego Franciotta, Marco Capobianco, Joseph Kuchling, Jürgen Haas, Mirjam Korporal-Kuhnke, Soeren Thue Lillevang, Kai Fechner, Kathrin Schanda, Friedemann Paul, Brigitte Wildemann, Markus Reindl, (2016) MOG-IgG in NMO and related disorders: a multicenter study of 50 patients. Part 1: Frequency, syndrome specificity, influence of disease activity, long-term course, association with AQP4-IgG, and origin. Journal of Neuroinflammation 13 (1) 10.1186/s12974-016-0717-1 PMC5084340 27788675

[33] Thorley-Lawson DA (2015). EBV Persistence-Introducing the Virus. Curr Top Microbiol Immunol.

